# Bridging gaps in the care of people under the age of 60 with complex impairments through cross-indication home-based rehabilitation: a cross-sectional survey with experts in Germany

**DOI:** 10.1186/s12913-026-15682-w

**Published:** 2026-09-24

**Authors:** Christoph Armbruster, Christian Schlett, Erik Farin-Glattacker

**Affiliations:** Section of Health Care Research and Rehabilitation Research, Institute of Medical Biometry and Statistics, Medical Faculty and Medical Center – University of Freiburg, 79106 Freiburg, Germany

**Keywords:** Rehabilitation, Health services accessibility, Home care services, Disabled persons, Needs assessment, Surveys and questionnaires, Germany

## Abstract

**Background:**

In Germany, medical rehabilitation services are generally well developed. However, first evidence suggests that there are care gaps in the current outpatient and inpatient indication-specific rehabilitation landscape, which may particularly affect people under 60 with complex impairments. Home-based rehabilitation could help to bridge these care gaps by providing access to rehabilitation for this group of patients. Currently, there are only a few such services for patients under 60. In this context, a cross-indication approach for needs-based care could be relevant to ensure adequate treatment of rehabilitation needs in their entirety. A systematic survey of a broad sample of experts in this field of research is still lacking. The aim of this exploratory study was therefore to assess the views of experts on (1) care needs of people under 60 with complex impairments that are insufficiently covered by current rehabilitation services, (2) the need and associated benefits of home-based rehabilitation as well as perceptions regarding a cross-indication approach, and (3) indication and allocation criteria for a cross-indication approach of home-based rehabilitation.

**Methods:**

We performed a cross-sectional online survey between 03/2024 and 05/2024 with experts from six professional fields from across Germany. We report descriptive statistics on experts’ assessments of a wide range of aspects of rehabilitation care and exploratively examined whether experts from different professional fields differ in their assessments.

**Results:**

A total of *n* = 136 experts took part in the survey. The majority of them were physicians in private practice. The experts tend to see a high level of unmet care needs in current rehabilitation services. Their positive assessment of home-based rehabilitation in terms of need and benefits, as well as their positive perception of a cross-indication approach, indicate that home-based rehabilitation conceptualised across all indications may help to bridge these care gaps.

**Conclusions:**

From the experts’ perspective, there are various care gaps in current rehabilitation services for people under 60 with complex impairments in Germany. As a complementary service, cross-indication home-based rehabilitation may be a promising but still empirically under-evaluated approach to optimise the functioning of this patient group.

**Trial registration:**

German Clinical Trials Register (DRKS): DRKS00029068; Date of registration: 28.06.2022.

**Supplementary Information:**

The online version contains supplementary material available at https://doi.org/10.1186/s12913-026-15682-w.

## Background

In the context of healthcare, rehabilitation (rehab) is a multimodal, person-centred, collaborative process that aims to optimise the functioning of people with health conditions currently experiencing disability or likely to experience disability, or persons with disability through a bundle of interventions, taking contextual factors into account [1]. According to the International Classification of Functioning, Disability, and Health (ICF), “functioning” refers to both physical and mental structures and functions, as well as to activities of daily living (e.g. washing oneself) and participation, i.e. a person’s involvement in everyday situations and society [2]. Rehab should be available and accessible for anyone who needs it [3] and should take place close to home, as required by the United Nations “Convention on the rights of persons with disabilities” (Article 26) [4].

Germany provides a comprehensive range of rehab and participation services, such as medical rehab, benefits for participation in working life (vocational rehab) or benefits for participation in community life [5]. This study focusses on medical rehab (hereafter referred to as rehab for simplicity), which is generally well developed in Germany [6]. The legal basis for rehab is mainly regulated in the Social Code V-VII and IX. There are several ways in which patients can be referred to rehab. Either via physicians in private practice [7] or as part of the care assessment by an assessor from the Medical Service [8]. Rehab can also follow a hospital stay (usually within 14 days) as so-called follow-up rehab [7, 9]. The costs of rehab are covered by various bodies, such as the German pension insurance or the statutory health insurance [10]. The majority of rehab services are provided on an inpatient basis and usually last 3 to 4 weeks [9]. This is due to historical reasons and a high availability of such centres [5]. In 2021, for instance, the proportion of rehab in inpatient centres within the German pension insurance was 83% [11]. Rehab can also take place in outpatient centres or in the patient’s familiar environment (e.g. at home or in a nursing home) - called home-based rehab (HbR) [9, 12]. Outpatient rehab services and HbR usually last a maximum of 20 treatment days [9, 13]. With regard to HbR, there are currently 28 centres, of which 27 rehabilitate geriatric patients [14]. For non-geriatric patients, defined in this study as people under the age of 60 [7], a neurological centre is available [15].

In Germany, HbR is based on a specific concept. It is delivered by a multiprofessional team (e.g. physiotherapists, occupational therapists, psychotherapists) under physician leadership, which works interdisciplinarily. Based on this concept and the diagnostics, the required interventions are defined jointly with the patient (and, if applicable, their relatives) and the team, which are reviewed through weekly team meetings, and adjusted as needed during the course of rehab [12, 13].

Rehab interventions for non-geriatric patients, provided on an inpatient, outpatient or HbR basis, follow an indication-specific approach and are therefore tailored to specific diseases [13, 16]. For example, rehab for someone who has had a stroke will take place in a centre with a neurological treatment focus [17]. In addition, the allocation to a specific setting should ideally be guided by where the rehabilitative goals can be most effectively achieved, given the strategies applicable in the corresponding setting. To illustrate, restitutive strategies can primarily be implemented in inpatient and outpatient settings to ensure the intensity and variety of interventions, supported by specialised equipment (e.g. for repetitive training in hydrotherapy pools or body-weight-supported treadmills). In contrast, adaptive strategies such as adaptations of assistive technologies typically require the patient’s familiar environment [13, 18].

The need for rehab is increasing worldwide [19, 20]. Factors such as improved medical care [21] or the growing prevalence of chronic diseases [20, 22] contribute to this trend. However, previous literature indicates the presence of care gaps in the rehab system, both nationally [e.g. 6, 23, 24] and internationally [19, 25, 26]. These gaps can lead to interruptions and an undersupply at various stages of the continuum of care such as after a hospital stay [6, 23, 25, 26]. In Germany, this can particularly affect non-geriatric patients with complex, usually multiple impairments [6, 23, 24, 27]. Complex impairments may result from severe acute events [28–31], from multimorbidity [32], which increasingly affects people under 60 [33–36], and also from contextual factors (e.g. language barrier in the case of a migration background) [27]. With regard to current outpatient and inpatient rehab services in Germany, this group of people - despite having a need for rehab and a positive rehab prognosis - does not always meet the requirements (i.e. lack of rehab capability due to insufficient physical or mental capacity, dependencies in performing everyday tasks such as washing oneself) to participate in rehab [37]. Specialised settings, such as inpatient neurological rehab, are an exception, as the requirement for rehab capability is less restrictive due to greater resources [18, 38]. Given the existing situation of care gaps worldwide, HbR in particular could play an important role in providing access to rehab at various stages of the continuum of care and thus contribute to an improvement in patients’ functioning [12, 25, 39]. Furthermore, and especially in the case of complex impairments, national and international recommendations [e.g. 23, 40, 41] suggest that rehab should be conceptualised on a cross-indication approach. In this case, rehab is organised inclusively, i.e. across the otherwise usual indication-specific areas [42]. As people with complex impairments, for whom a positive rehab prognosis can only be expected with the involvement of their familiar environment, are the main target group of HbR [12, 43], such an approach is considered particularly important in this type of care [43, 44]. This should ensure adequate treatment of the patients’ complex impairment profile and the resulting rehab needs in their entirety [23, 24, 29], and at the same time prevent the rehab interventions from focussing exclusively on a rehab-induced main diagnosis [44, 45]. In this context, the term “cross-indication” means that the rehab team addresses common impairments that may be equally present at the levels of physical functions, activities and participation (e.g. impairments in mobility, communication, activities of daily living) for different diagnoses or multimorbidity. Similar to geriatric rehab, the function-, activity- and participation-orientated team can therefore care for patients from different indication areas simultaneously [12, 46, 47].

Up to now, knowledge about care gaps for non-geriatric patients in the German rehab system, the need for HbR including perceptions of a cross-indication approach, and potential indication and allocation criteria for a cross-indication approach of HbR has only been based on experience reports and conceptual considerations [e.g. 23, 44] or has been qualitatively researched among a small number of experts [43]. Thus, it remains unclear to what extent these views (e.g. on current care gaps) are widely shared. The aim of this study was therefore to assess the views of a broad sample of relevant experts directly related to rehabilitative processes on (1) care needs of people under 60 with complex impairments that are insufficiently covered by current rehab services (i.e. outpatient and inpatient indication-specific centres), (2) the need and associated benefits of HbR for this group of people as well as perceptions regarding a cross-indication approach, and (3) indication and allocation criteria for a cross-indication approach of HbR.

## Methods

We report this study using the Checklist for Reporting of Survey Studies (CROSS) [48]. The study was registered in the German Clinical Trials Register (DRKS00029068) and approved by the ethics commission of the Albert-Ludwigs-University in Freiburg (Approval No. 22-1200-S2).

### Instrument

This study was conducted in the form of a cross-sectional online survey with different groups of experts directly involved in rehab processes. For the online survey, we developed a questionnaire based on the findings of a preliminary qualitative interview study with 22 experts [43]. In a first step, an initial pool of 75 items (partly similar in content but differing in terms of sentence structure) was created and mapped by topic alongside the corresponding interview excerpts in a structured table to ensure the traceability of each item to the qualitative data. When formulating the items, we paid particular attention to aligning the terminology with the experts’ linguistic and professional context [49]. Next, we discussed the items with two experts with in-depth knowledge of the topic in order to make initial linguistic adaptations and to create a representative selection of items in terms of content validity (46 items were removed, 17 refined, and 2 added) [50]. In addition, we developed a characterisation of the target group “people under 60 with complex impairments and rehab needs” to ensure a standardised assessment of the items (see Box 1) [51]. Due to the heterogeneity of this group of people and the variability of characteristics from person to person [52], we did not provide any further instructions regarding which aspects the experts should consider in their assessments.

**Box 1:** Characterisation of people under 60 with complex impairments and rehab needs

For example, it may be that
1. there is a high need for care (including treatment care). E.g. open wounds, complex orthoses etc.
2. there is a single or multiple disability (sensory, motor, communicative, psychological) that makes it impossible to be independent on the ward and/or to participate independently in group therapies. E.g. blindness, cerebral palsy, etc.
3. there is a particularly high individual need for individual therapies. E.g. polytrauma, cognitive impairments, etc.
4. there is a need for treatment of one or more conditions (multimorbidity) which cannot be addressed by the indication-specific rehab interventions or with reasonable effort. E.g. orthopaedic and cardiological and/or psychotherapeutic indication, etc.
5. there are certain contextual factors that make it impossible or impractical to be away from home. E.g. responsibility for children, language barrier in the case of a migration background, etc.
6. environmental and personal factors in one’s own social environment need to be strengthened (adaptive rehab strategy) in order to achieve individual goals. E.g. adaptations of assistive technologies in one’s own home, etc.

Note: The experts were informed that mixed forms of these aspects are also possible

The first draft of the questionnaire contained a total of 31 items with two overarching topics, i.e. “current rehabilitative care situation” and “HbR”. The first topic was divided into “rehab services and access” and “home care and nursing homes”. The second topic included items on “HbR in general”, on a “cross-indication approach” and an item on “indication and allocation criteria”, in which 24 diagnoses/circumstances were asked about their importance for a cross-indication approach. With the exception of the latter, the items were provided with a 6-point Likert scale from 1 = “does not apply at all” to 6 = “totally applies”. Regarding the indication and allocation criteria, the diagnoses/circumstances were provided with the response categories 1 = “not at all important”, 2 = “somewhat important” and 3 = “very important”. Assuming that certain expert groups may not have experience or knowledge in all areas, we have added the category “I cannot judge” to all items to counteract possible frustration [53]. Furthermore, we included 5 items to collect sociodemographic characteristics of the respondents. Subsequently, we conducted a pretest in written form with three researchers and three experts from the field (some of them potential participants). In addition to questions on structure, spelling and processing time [51, 54], we also carried out a comprehension probing [55]. Feedback, e.g. on the wording of individual introductions to a new topic, was finally incorporated. To the total of 36 items (31 + 5), no further items were added. The entire questionnaire was originally developed in German, and all results are based on this German-language instrument. For the purpose of this article, we translated the questionnaire into English.

### Study design and recruitment

The online survey was conducted between 03/2024 and 05/2024 using the REDCap software (version 14.0.30) [56, 57]. We recruited experts from six professional fields directly related to rehab processes from across Germany (see Box 2). The reason for including these expert groups was to be able to capture experience both before and during rehab. In addition, we have included experts with experience of cross-indication concepts (i.e. physicians or senior therapists working in cross-indication early rehab settings or geriatric HbR) [44]. For recruitment, different strategies were used: (1) Invitation through gatekeepers of organisations and institutions via mailing lists (e.g. Institute of General Practice at the Medical Center - University of Freiburg), (2) Direct distribution of the invitation via publicly accessible email addresses, (3) Call for participation via various homepages and newsletters (e.g. German Association for Rehabilitation), and (4) Information flyers with QR code (e.g. distributed at a symposium on HbR). For recruitment strategies (1) and (2), we reminded potential participants of the online survey again at a later date. Participants were directed to the online survey via a link. Prior to the actual survey, written information was provided and explicit consent for data collection and analysis was obtained. After consent, the inclusion criteria were queried (s. Box 2). If no consent was given or if the participant did not belong to one of the expert groups, the survey was automatically terminated. Due to the exploratory study design, no sample size calculation was carried out a priori. However, we aimed for a sample size of *n* > 100 for the survey period in order to perform psychometric analyses such as factor analyses and reliability analyses on item responses to explore and evaluate possible factorial structures [58, 59].

**Box 2:** Inclusion criteria

(A) Allocation/initiation of rehab:
1. Physicians or senior therapists in cross-indication early rehab settings (i.e. hospitals)
2. Physicians in private practice (i.e. general practitioners or specialists in physical medicine and rehab)
3. Discharge managers in hospitals
4. Assessors from the Medical Service or Socio-Medical Service
(B) Provision of rehab interventions:
5. Physicians or senior therapists in (geriatric) home-based rehab
6. Physicians in indication-specific rehab centres (neurology, cardiology, pneumology and orthopaedics)

### Data analysis

First, we checked the data for plausibility. Descriptive statistics were used to analyse the data at item level. In order to identify items that participants perceived as very similar in terms of content and therefore can be summarised into a single scale value [60], we performed an exploratory factor analysis (EFA) within the respective topic areas of the questionnaire “rehab services and access”, “HbR in general” and “cross-indication approach”, according to respective best practice recommendations [61]. The number of factors was determined using the scree plot, parallel analysis and the consideration of model fit criteria such as Velicer’s Minimum Average Partial (MAP) criterion. We constructed scales if ≥4 items per factor were clearly interpretable. The unidimensionality of each scale was then tested using confirmatory factor analysis (CFA). To estimate reliability, we computed McDonald’s omega. If ≥75% of the items per scale were answered, scale values were calculated by averaging the item responses. We then exploratively calculated the bivariate correlations between the scale values to determine the direction and strength of the relationships (e.g. between the unmet care needs in the current rehab services and the general assessment of HbR).

Furthermore, we exploratively examined group differences for the variables “professional field” and “knowledge of HbR”. For the variable “professional field”, we used the Kruskal-Wallis test, as one group contained *n* < 10 cases [62]. Post-hoc tests were performed using the Wilcoxon rank-sum test for unpaired samples. Group differences on the variable “knowledge of HbR” were calculated using an unpaired t-test. We report median with IQR for non-parametric tests and mean with SD for parametric tests. With the exception of the 24 diagnoses/circumstances of the topic “indication and allocation criteria”, the scales identified in the EFA and the remaining single items were used as dependent variables. The significance level was set at *p* < 0.05. Due to the exploratory approach, we did not apply a p-value adjustment [63]. For all the analyses we used R (version 4.2.2) [64] with the packages psych [65], lavaan [66], MBESS [67] and rstatix [68].

## Results

### Response behaviour and sample characteristics

A total of *n* = 235 people visited the survey website, of which *n* = 189 (80.4%) agreed to participate in the study. Of these, up to *n* = 144 (76.2%) started to answer the first section of the questionnaire (i.e. “rehab services and access”). A small number of participants dropped out during the course of the survey (*n* = 8), resulting in a final sample size of *n* = 136 (72.0%). In terms of sample characteristics, the 50 to under 60 age group constituted the majority with 41.9%. The sample consisted of slightly more women (56.7%) than men. Most of the participants were physicians in private practice (28.7%), of which 71.1% were general practitioners. Almost three-quarters of respondents had more than 15 years of work experience. About half of the participants (46.7%) stated that they had no knowledge of HbR prior to the online survey. Table 1 shows the characteristics of the sample.

**Table 1:** Sample characteristics

	*n*	%^g^
**Age**		
until below 30	9	6.6
30 to under 40	20	14.7
40 to under 50	17	12.5
50 to under 60	57	41.9
60 and older	32	23.5
no specification	1	0.7
**Gender**		
female	76	56.7
male	56	41.8
no specification	2	1.5
**Professional field**		
Physician or senior therapist in (geriatric) HbR^a, d^	15	11.0
Physician or senior therapist in cross-indication early rehab setting (i.e. hospital)^c^	11	8.1
Physician in indication-specific rehab centre^d, e^	34	25.0
Physician in private practice^c, f^	39	28.7
Discharge manager in hospital^c^	24	17.6
Assessor from the Medical Service or Socio-Medical Service^c^	5	3.7
no specification	8	5.9
**Work experience** (in years)^b^		
until below 2	2	1.5
2 to under 5	10	7.4
5 to under 10	9	6.6
10 to under 15	18	13.2
15 and more	96	70.6
no specification	1	0.7
**Knowledge of HbR**		
no	63	46.7
yes	72	53.3

^a^ Home-based rehabilitation

^b^ Related to the specified professional field

^c^ Allocation/initiation of rehab

^d^ Provision of rehab interventions

^e^ Specification by *n* = 33, i.e. neurology *n* = 4 (12.1%), cardiology *n* = 2 (6.1%), pneumology *n* = 4 (12.1%), orthopaedics *n* = 23 (69.7%)

^f^ Specification by *n* = 38, i.e. general practitioner *n* = 27 (71.1%), specialist in physical medicine and rehab *n* = 11 (28.9%)

^g^ Data refer to valid cases

### Description of the scales

The EFAs resulted in the following factors: On the topic of “rehab services and access” (11 items in total), we were able to identify a meaningful factor with 8 items. The test for unidimensionality using a CFA showed an acceptable model fit (CFI = 0.93, TLI = 0.93, SRMR = 0.08), albeit the Chi²-test was significant (Chi²(27) = 45.13, *p* = 0.016). McDonald’s omega was ω = 0.86, 95% confidence interval (CI) [0.79, 0.92], which indicates a high inter-item correlation [69]. Conceptually, the scale can be defined as “unmet care needs” and ranges from 1 to 6. A high value indicates a high level of unmet care needs in the current rehab services (i.e. indication-specific outpatient and inpatient centres) for people under 60 with complex impairments. In relation to the topic of “HbR in general”, all 11 items could be summarised into one factor. The model fit criteria within the CFA partly indicate an insufficient fit, i.e. significant Chi²-test with Chi²(44) = 107.81 (*p* < 0.001), CFI = 0.89, TLI = 0.86, SRMR = 0.05. McDonald’s omega was ω = 0.93, 95% CI [0.90, 0.97]. We interpret the scale as “general assessment of HbR”. The higher the value (from 1 to 6), the more positive the assessment of HbR in terms of need and benefits. It was also possible to summarise the 4 items of the topic “cross-indication approach” into one factor. Again, the model fit criteria within the CFA partly indicate an insufficient fit, i.e. significant Chi²-test with Chi²(2) = 11.88 (*p* = 0.003), CFI = 0.94, TLI = 0.82, SRMR = 0.05. McDonald’s omega was ω = 0.90, 95% CI [0.84, 0.97]. We label the scale as “cross-indication approach”. The higher the value (from 1 to 6), the more positive the perception of a cross-indication approach within HbR in the care of people under 60 with complex impairments. The corresponding items of the respective scales are presented in Table 2.

**Table 2:** Scales and descriptive statistics on the topics of the questionnaire (excluding “indication and allocation criteria”)

Topics/Items		*n* ^a^	M^b^ (SD)^c^	Agreement (%)^d^
**Rehab services and access**				
Scale: Unmet care needs		123	4.65 (0.87)	
1.	Despite the need for rehabilitation, this group of people often lack sufficient capability to participate in rehabilitation.	136	3.97 (1.31)	62.5
2.	There are too few outpatient and inpatient indication-specific rehabilitation centres that can deal with the high need for support of this group of people.	140	5.02 (1.12)	89.3
3.	Due to the lack of appropriate rehabilitation services for this group of people, other options (e.g. neurological rehabilitation or inpatient geriatric rehabilitation) are sometimes used.	136	4.79 (1.10)	89.0
4.	For this group of people, follow-up rehabilitation in an indication-specific rehabilitation centre is often not feasible after acute inpatient care.	132	4.56 (1.15)	86.4
5.	This group of people can hardly benefit from classic group therapy (e.g. circuit training) provided by outpatient and inpatient indication-specific rehabilitation centres.	133	4.62 (1.25)	84.2
6.	For this group of people, a longer overall duration of rehabilitation is needed in order to focus the rehabilitation on participation.	135	5.01 (1.09)	91.9
7.	For this group of people, allocation to a specific rehabilitation area is difficult.	130	4.63 (1.11)	84.6
8.	Rehabilitation interventions in indication-specific outpatient and inpatient rehabilitation centres do not sufficiently cover other important needs of this group of people in addition to the main indication.	132	4.53 (1.14)	83.3
Further aspects of care^e^				
9.	The therapeutic success achieved in outpatient rehabilitation centres can be better transferred to the familiar environment (e.g. own home) than in inpatient rehabilitation centres.	129	3.90 (1.39)	61.2
10.	In indication-specific outpatient and inpatient rehabilitation centres, the cross-indication needs of this group of people can be clarified very well consultatively.	125	3.30 (1.24)	40.0
11.	In outpatient and inpatient indication-specific rehabilitation centres, accompanying persons (e.g. relatives) are not sufficiently involved in the treatment concept.	122	4.11 (1.08)	71.3
**Home care and nursing homes**				
1.	Despite the need for rehabilitation, this group of people is often discharged to another setting (e.g. their own home or a nursing home) after acute inpatient care.	132	4.64 (1.01)	87.9
2.	For this group of people, the need for rehabilitation cannot (completely) covered by the established outpatient services of occupational-, speech- or physiotherapists in private practices or other allied health professionals.	135	4.90 (1.07)	92.6
**HbR** ^f^ ** in general**				
Scale: General assessment of HbR		123	4.93 (0.83)	
1.	Care deficits in outpatient and inpatient indication-specific rehabilitation centres can be avoided by home-based rehabilitation for this group of people.	126	4.45 (1.24)	84.9
2.	In order to care for this group of people, home-based rehabilitation services need to be expanded nationwide.	129	5.00 (1.17)	89.9
3.	Home-based rehabilitation is important for this group of people in order to enable follow-up rehabilitation after acute inpatient care.	126	4.74 (1.16)	86.5
4.	Home-based rehabilitation is important in order to enable rehabilitation interventions despite an existing bacterial colonisation (e.g. MRSA) of the affected person.	134	4.82 (1.21)	87.3
5.	Home-based rehabilitation can help to prepare this group of people for subsequent rehabilitation in an indication-specific rehabilitation centre.	132	4.77 (1.07)	89.4
6.	After rehabilitation in an indication-specific rehabilitation centre (if this could be realised), subsequent home-based rehabilitation should be possible for this group of people.	131	4.84 (1.05)	90.8
7.	Home-based rehabilitation has the advantage for this group of people that the individual everyday problems in the social environment (e.g. at home, at work) are directly visible and can be addressed during the rehabilitation.	132	5.31 (0.82)	97.7
8.	Home-based rehabilitation has the advantage for this group of people that the therapy can be very individualised.	134	5.01 (1.18)	90.3
9.	Home-based rehabilitation has the advantage for this group of people that the whole rehabilitation process can be extended over a longer period.	122	4.84 (1.12)	90.2
10.	During home-based rehabilitation, it is very important to involve the social environment (e.g. relatives) in the treatment concept.	134	5.37 (0.76)	99.3
11.	Rehabilitation in the familiar environment is more likely to be accepted by this group of people and possibly their relatives.	109	4.71 (1.17)	87.2
**Cross-indication approach**				
Scale: Cross-indication approach		116	5.02 (0.94)	
1.	Home-based rehabilitation should be conceptualised on a cross-indication approach for the care of this group of people.	133	5.09 (1.00)	95.5
2.	Cross-indication home-based rehabilitation contributes to a more holistic view of this group of people than an indication-specific approach.	131	5.07 (1.12)	94.7
3.	Cross-indication home-based rehabilitation facilitates allocation in the case of interdisciplinary issues.	124	4.95 (1.08)	90.3
4.	Cross-indication home-based rehabilitation can provide care for a larger group of people in a given catchment area than an indication-specific approach.	124	4.83 (1.19)	91.1

^a^ Valid cases

^b^ Mean (response categories: 1 = “does not apply at all”, 2 = “does not apply”, 3 = “does rather not apply”, 4 = “tends to apply”, 5 = “applies”, 6 = “totally applies”)

^c^ Standard deviation

^d^ Percentage “tends to apply” to “totally applies”

^e^ Items not included in the scale “unmet care needs”

^f^ Home-based rehabilitation

### Current rehabilitative care situation

#### Rehab services and access

Table 2 provides an overview of the descriptive statistics for each topic of the questionnaire (excluding “indication and allocation criteria”). Regarding the topic “rehab service and access”, the experts tend to see a high level of unmet care needs in the current rehab services for this group of people (scale values: *n* = 123, M = 4.65, SD = 0.87). For ease of interpretation, the following results on the item level are presented as the percentage of experts who chose a response expressing agreement with the statement. The corresponding means and SDs are shown in Table 2. A total of 62.5% of the experts thought that this group of people, despite their need for rehab, were not sufficiently able to participate in rehab. In some cases, other options (e.g. neurological rehab) have to be used to realise a rehab (89.0%), and there are too few rehab centres able to deal with this group of people (89.3%). Furthermore, experts saw problems regarding group therapy, allocation, the duration of rehab, as well as addressing cross-indication needs (approx. 83–92%). In terms of group differences, experts working in discharge management or as assessors for the Medical Service or the Socio-Medical Service reported the highest level of unmet care needs (i.e. each with a median of 5.12 and an IQR of 0.75 and 0.25 respectively). In contrast, participants from the field of cross-indication early rehab showed the lowest level of unmet care needs with a median of 4.31 (IQR = 1.06) and differed significantly from all other groups. With regard to the assessment of the transfer of the therapy success to the familiar environment (further aspects of care), there were clear differences between the groups. In particular, experts working in (geriatric) HbR differed significantly from all other groups due to a very high level of agreement (Mdn = 6.00, IQR = 1.00). Experts with prior knowledge of HbR also differed significantly from those who were not yet familiar with HbR (M = 4.28, SD = 1.46 and M = 3.45, SD = 1.25, respectively). A detailed overview of the group differences, corresponding significance tests and associated effect sizes can be found in Additional file 1.

#### Home care and nursing homes

Most of the experts (87.9%) stated that affected people are often discharged to their own home or transferred to a nursing home after a hospital stay, even though they require rehab. According to the respondents, the provision of established outpatient services (e.g. physiotherapy in private practice) is not sufficient to cover the existing need for rehab (92.6%). No significant group differences could be identified within this topic (see Additional file 2).

### Home-based rehab

#### Home-based rehab in general

Experts rated HbR mostly positive in terms of need and benefits for the care of people under 60 with complex impairments (scale values: *n* = 123, M = 4.93, SD = 0.83). As regards the individual items, most experts rated that HbR should be expanded nationwide (89.9%). HbR can also help to bridge the gaps in existing rehab services for this group of people (84.9%) and enable follow-up rehab after hospital stay (86.5%). The advantages of HbR were also seen in preparing this group of people for subsequent rehab (e.g. in an inpatient centre), identifying barriers in the social environment (e.g. at home or at work) and organising the rehab process in a flexible way (e.g. rehab activities mainly at times when the person is physically fit) (approx. 89–98%). Many experts (87.2%) also thought that HbR is more likely to be accepted by this group of people and possibly their relatives. In terms of group differences, experts working in (geriatric) HbR or in discharge management were most positive about HbR (Mdn = 5.45, IQR = 0.49 and Mdn = 5.25, IQR = 0.63, respectively), and in most cases differed significantly from all other groups. Experts with and without prior knowledge of HbR did not differ in their positivity regarding the need and benefits of HbR (M = 4.99, SD = 0.99 and M = 4.87, SD = 0.58, respectively). For more details on group differences, see Additional File 3.

#### Cross-indication approach

The experts considered a cross-indication conception of HbR to be positive (scale values: *n* = 116, M = 5.02, SD = 0.94). Compared with an indication-specific HbR, participants saw advantages in easier allocation in the case of cross-indication needs, more holistic rehab and the possibility of being able to care for a larger group of people within a catchment area (approx. 90–95%). There were no significant group differences on the scale in relation to the professional field and previous knowledge of HbR (see Additional file 4). Several experts (*n* = 22) also gave further reasons why a cross-indication HbR seems appropriate (free text question). Comments were mainly about holistic care. According to them, cross-indication HbR would better address the often multiple needs of this group of people, be more ICF oriented, contribute to interdisciplinary care, and reduce the number of different outpatient therapies in various locations. This approach would also be more resource-efficient, as multiple indications could be covered by one HbR-team, and it would help to reduce the burden on other service providers, such as physicians in private practice.

### Bivariate correlations of the scales

The bivariate correlations showed that the expert assessment of the extent of unmet care needs correlated moderately positively with the general assessment of HbR (*r* = 0.36, 95% CI [0.19, 0.52]) and with the positive perception of a cross-indication approach (*r* = 0.31, 95% CI [0.12, 0.47], while the scales of the latter two assessments correlated highly positively with each other (*r* = 0.71, 95% CI [0.63, 0.80]). All correlations were significant with *p*≤0.001.

### Indication and allocation criteria

Table 3 shows the ratings for the 24 diagnoses/circumstances for which a cross-indication approach of HbR would be important. We pointed out in the survey that these diagnoses/circumstances are not necessarily to be considered separately as justifying rehab, but may also be present in variable combinations (i.e. comorbidity or multimorbidity). Diagnoses/circumstances that were rated as “very important” by > 60% of the experts were from the following areas: (1) Mental functions (i.e. perceptual and cognitive disorders, dementia, anxieties, post-traumatic stress disorder, depression); (2) Cardiovascular and respiratory system functions (i.e. reduced cardiopulmonary capacity (e.g. due to coronary heart disease (CHD), chronic obstructive pulmonary disease (COPD)); (3) Neuromusculoskeletal and movement-related functions (i.e. amyotrophic lateral sclerosis, stroke, high spinal cord injury, polytrauma, immobility); (4) Congenital or acquired disabilities (i.e. blindness); (5) Need for treatment care (e.g. drainages, bladder catheters, artificial respiration, percutaneous endoscopic gastrostomy (PEG)). Some participants (*n* = 17) provided information on other diagnoses/circumstances (free text question). Deficits in the area of neuromusculoskeletal and movement-related functions were mentioned most frequently, such as Parkinson’s disease (2x), polyneuropathy (2x), muscular dystrophy, Guillain-Barré syndrome, and myasthenia gravis.

**Table 3:** Indication and allocation criteria

Diagnoses/Circumstances^a^		*n* ^b^	Mdn^c^ (IQR)^d^	not at all important (%)	somewhat important (%)	very important (%)
1.	Perceptual and cognitive disorders	128	3 (0)	2 (1.6)	28 (21.8)	98 (76.6)
2.	Wandering	107	3 (1)	6 (5.6)	42 (39.3)	59 (55.1)
3.	Delirium	115	3 (1)	13 (11.3)	34 (29.6)	68 (59.1)
4.	Dementia	124	3 (1)	12 (9.7)	22 (17.7)	90 (72.6)
5.	Anxieties	127	3 (1)	2 (1.5)	34 (26.8)	91 (71.7)
6.	Post-traumatic stress disorder	121	3 (1)	5 (4.1)	41 (33.9)	75 (62.0)
7.	Schizophrenia	111	3 (1)	9 (8.1)	48 (43.3)	54 (48.6)
8.	Personality disorders	117	2 (1)	8 (6.8)	58 (49.6)	51 (43.6)
9.	Depression	130	3 (1)	5 (3.8)	47 (36.2)	78 (60.0)
10.	Reduced cardiopulmonary capacity (e.g. due to coronary heart disease (CHD), chronic obstructive pulmonary disease (COPD))	131	3 (1)	10 (7.6)	41 (31.3)	80 (61.1)
11.	Chronic wounds (e.g. due to peripheral arterial disease (PAD))	131	3 (1)	9 (6.9)	56 (42.7)	66 (50.4)
12.	Multiple sclerosis	127	3 (1)	7 (5.5)	51 (40.2)	69 (54.3)
13.	Amyotrophic lateral sclerosis	117	3 (1)	4 (3.4)	34 (29.1)	79 (67.5)
14.	Stroke	130	3 (1)	6 (4.6)	35 (26.9)	89 (68.5)
15.	High spinal cord injury	125	3 (1)	6 (4.8)	27 (21.6)	92 (73.6)
16.	Polytrauma	125	3 (1)	2 (1.6)	31 (24.8)	92 (73.6)
17.	Fractures	127	2 (1)	14 (11.0)	56 (44.1)	57 (44.9)
18.	Chronic pain syndrome	129	3 (1)	12 (9.3)	40 (31.0)	77 (59.7)
19.	Blindness	126	3 (1)	7 (5.6)	42 (33.3)	77 (61.1)
20.	Spina bifida	113	2 (1)	9 (8.0)	49 (43.4)	55 (48.6)
21.	Infantile cerebral palsy	116	3 (1)	7 (6.0)	41 (35.4)	68 (58.6)
22.	Intellectual disability	125	3 (1)	7 (5.6)	54 (43.2)	64 (51.2)
23.	Need for treatment care (e.g. drainages, bladder catheters, artificial respiration, PEG^e^)	130	3 (1)	8 (6.2)	38 (29.2)	84 (64.6)
24.	Immobility	130	3 (1)	7 (5.4)	32 (24.6)	91 (70.0)

^a^ Note in the survey that diagnoses/circumstances can also occur in different combinations (i.e. comorbidity or multimorbidity) and should not necessarily be considered separately

^b^ Valid cases

^c^ Median

^d^ Interquartile range

^e^ Percutaneous endoscopic gastrostomy

## Discussion

To the best of our knowledge, this is the first quantitative study, involving various relevant experts directly related to rehab processes, that has investigated (1) insufficiently covered rehab needs of people under 60 with complex impairments in current rehab services, (2) the need and associated benefits of HbR as well as perceptions regarding a cross-indication approach, and (3) indication and allocation criteria for a cross-indication approach of HbR. Overall, the results show that the experts tend to see a high level of unmet care needs in current rehab services in Germany. Their positive assessment of HbR in terms of need and benefits, as well as their positive perception of a cross-indication approach, indicate that HbR conceptualised across all indications may help to bridge these care gaps.

With regard to the current outpatient and inpatient indications-specific rehab services, the respondents stated that, despite the need for rehab, there may be a lack of sufficient rehab capability. This finding is in line with previous literature [6, 23, 26, 37]. In this context, it has been criticised that the construct of rehab capability in Germany is too exclusionary, especially for people with complex impairments who are dependent on assistance for everyday tasks, and should therefore be reconsidered [37]. The construct primarily refers to the (often limited) staff resources and available therapy provision (e.g. primarily group therapy) of a rehab centre, rather than to an individual’s capability to participate in a suitable rehab programme [37]. Similar criticism has been expressed by authors from other countries. In practice, it is sometimes prematurely assumed that patients have no rehab potential, resulting in no rehab taking place, whereas the real problem is rather a lack of staff resources or knowledge in dealing with this patient group [70, 71]. According to our findings, this also explains why there is a perceived shortage of rehab centres with appropriate staff resources, which is also described outside the German rehab landscape [72, 73]. For example, Gupta et al. [72] demonstrated that the rehab workforce is particularly scarce in low-income countries, with less than 0.5 professionals per 10.000 inhabitants. The challenge of staff shortage also becomes apparent when patients are discharged from hospital to their own home or a nursing home instead of undergoing follow-up rehab. Beyer and Seidel [21] already pointed out this problem in 2017, which still exists despite proposed solutions [6, 23]. Additionally, the increasing pressure of shorter hospital stays, reflected in a reduction from 9.20 to 7.24 days between 2000 and 2019 across 25 European countries [74], can further exacerbate this situation. The finding that people with complex impairments can hardly benefit from group therapy was also shown by qualitative results from a recently published study of *n* = 733 people affected by long COVID or post-COVID syndrome in the context of inpatient rehab [75]. Language barriers, such as those experienced after a stroke, can further limit the active participation in group therapy [76]. Nevertheless, group therapy is associated with significant benefits, including the opportunity to share experiences, reduce feelings of isolation, and increase motivation to engage actively in rehab [77, 78]. Consequently, rehab interventions should be tailored to the individual needs of each patient and funded accordingly. Furthermore, respondents also felt that the length of rehab (usually 3 to 4 weeks inpatient, maximum 20 treatment days outpatient) is too short to provide rehab in a participation-oriented way. In this context, a RCT showed that for *n* = 53 patients with acquired brain injuries after inpatient rehab, a long-term outpatient rehab programme that included home visits was significantly superior to the standard outpatient treatment (e.g. physiotherapy) after 4 weeks in terms of achieving individual participation goals [17]. This demonstrates that patients still have rehab potential even after the usual rehab period. Our results also indicate that a consultative clarification of cross-indication needs (i.e. rehab needs that go beyond the main indication) is not optimally achieved in indication-specific rehab centres. However, it has already been described that the conditions for inpatient centres are actually good, as 48% of the 876 centres examined provide expertise in two and 26% in three indication areas [16]. Future research should investigate whether the challenge of a consultative clarification is due to the type of consultation (i.e. seeking expertise from external actors such as general practitioners vs. seeking expertise from colleagues within a rehab centre) and/or the type of the rehab centres (i.e. outpatient centre vs. inpatient centre).

The finding that HbR may play an important role for people under 60 with complex impairments due to existing care gaps is consistent with the existing literature [6, 23, 24, 43]. Experts working in the field of HbR were most positive about HbR in terms of need and benefits and differed significantly from the other expert groups (with the exception of the discharge managers). This could be explained by the positive experiences in their daily work [43, 47]. Evidence from a meta-analysis comprising 49 studies supports this, illustrating that independence in everyday tasks could be significantly improved in stroke survivors through HbR [79]. Respondents also see advantages in the flexibility (continuum of care), the individualisation, and in the possibility of involving the social environment (e.g. at home, at work) in the rehab process, making potential barriers directly visible. Particularly for people of working age, it can be important to consider the work environment early in the rehab process. A study regarding patients with spinal cord injuries has shown that one of the current challenges for occupational participation is the lack of transition from rehab into working life [80]. Moreover, according to our results, HbR could be more likely to be accepted by people under 60 with complex impairments and may therefore lead to a higher utilisation of rehab. This finding is consistent with previous studies showing that the utilisation of rehabilitative services can depend on individual or contextual factors (e.g. the desire not to leave the familiar environment) [43, 47, 79, 81]. However, a previous qualitative review found that while HbR provides a sense of privacy by allowing patients to train in their familiar environment, it can also lead to a perceived loss of privacy when aspects of their home life become visible to others. Additionally, it has been demonstrated that the implementation of HbR may be affected by a lack of resources such as supportive family members [25].

Furthermore, the experts perceived a cross-indication approach of HbR as positive, which is in line with recently published conceptual considerations [44] and the results of a qualitative study [43]. In the post-acute German rehab landscape, cross-indication concepts have so far only been established in geriatric care. It has already been shown that such concepts can also work for non-geriatric patients [42, 82]. Nevertheless, an exclusively cross-indication approach of HbR has been criticised by some rehab practitioners, who argue that it could hinder the achievement of challenging goals when multiple indications are combined. In their view, indication-specific approaches therefore remain important [43]. On the other hand, according to our findings, the experts also stated that a cross-indication approach could facilitate the allocation of patients, which illustrates that this is a current challenge in practice (see also the topic “rehab services and access”). To facilitate the allocation, the implementation of a superordinate instance in hospitals, that accompanies the rehab process from the beginning, has already been called for as an important strategy for follow-up rehab [83]. In this context, specialists in physical medicine and rehab could provide comprehensive expertise and support physicians with less rehabilitative expertise in an advisory function [26, 84]. In general, specialists in physical medicine and rehab are considered crucial to strengthening rehab services and responding adequately to both the increasing national and international needs for rehab. This, however, requires expanded academic resources, as the growth of the speciality as an academic discipline is fundamentally linked to its presence in medical faculties [85–87].

The assessment of the indication and allocation criteria can be understood as a comprehensive overview where the experts consider a cross-indication approach of HbR as important. It can be assumed that a large number of the aspects investigated, such as chronic obstructive pulmonary disease, are often accompanied by additional chronic health conditions [32]. The current recommendations for HbR in Germany describe similar diagnoses and circumstances, but in the context of an indication-specific approach [13]. We therefore suggest that these findings should be taken into account in the further development of HbR. In addition, it should be analysed which common impairments can be identified within these aspects in order to derive appropriate rehab strategies.

### Strengths and limitations

One of the strengths of this study is that we surveyed a wide range of experts with different roles in the rehab process across Germany in order to capture different perspectives and ensure a comprehensive understanding of this research topic. Furthermore, we developed the questionnaire based on the findings of a preliminary qualitative study and with the involvement of experts in this field of research in a multistage process. However, one limitation is that the results are not based on objective data (e.g. routine data), but on an experience-based assessment by the experts. It should also be noted that the formation of scale values in our study primarily served to structure and summarise the results, and that their use as measurement instruments in future studies would require further psychometric evaluation. In this context, particularly for scales showing only partial fit in the CFAs, the results on the item level may provide useful additional information beyond the scale values. The fact that nearly half of the experts were unaware of HbR prior to the online survey represents an additional limitation. Although a characterisation of people under 60 with complex impairments was provided (Box 1), experts may still have differed in their interpretation of the target population due to their different backgrounds and experiences, which may have introduced some degree of response bias. Another limitation is that the results are specific to the German rehab system, which reduces their applicability to other countries where rehab services are primarily provided on an outpatient basis [88]. Furthermore, despite conducting a survey across Germany, the specific regions where the experts were located were not recorded. In addition, the small number of cases in certain expert groups limited the generalisability of subgroup-specific findings and reduced the precision and statistical power of the comparative analyses. Thus, existing differences between the groups may have remained undetected and observed differences may be less stable. Given our exploratory approach, it should also be noted that we did not apply a p-value adjustment in the statistical tests, which may have increased the likelihood that differences between expert groups that arose by chance were identified as statistically significant. Moreover, the study design introduces further limitations, including a potential selection bias and the omission of therapeutic perspectives from indication-specific rehab centres, which may limit the comprehensiveness of the results. Consequently, these findings need to be interpreted with caution.

## Conclusions

In summary, the results show that experts tend to see a high level of unmet care needs in the current rehab services for people with complex impairments under the age of 60 in Germany. Their perception also suggest that HbR may help to bridge these care gaps and should therefore be expanded. In this context, the experts consider a cross-indication approach to be necessary. As a complementary rehab service to the current outpatient and inpatient indication-specific centres, cross-indication HbR may be a promising but still empirically under-evaluated approach to optimise the functioning of this patient group. Conceptual developments are therefore required. Future research could then focus on patients’ and/or relatives’ perspectives on cross-indication HbR, on effects on the level of functioning (e.g. changes in health, participation, and disability), or on cost-benefit analyses.

## Supplementary Information

Below is the link to the electronic supplementary material.

Supplementary Material 1: Additional file 1 Group differences on the topic “rehab services and access”

Supplementary Material 2: Additional file 2 Group differences on the topic “home care and nursing homes”

Supplementary Material 3: Additional file 3 Group differences on the topic “HbR in general”

Supplementary Material 4: Additional file 4 Group differences on the topic “cross-indication approach”

## Data Availability

The datasets generated and/or analysed during the current study are not publicly available due due to data protection agreements within the project but are available in anonymised form from the corresponding author upon reasonable request.

## Abbreviation

CFA: Confirmatory factor analysis
CI: Confidence interval
EFA: Exploratory factor analysis
HbR: Home-based rehabilitation
ICF: International Classification of Functioning, Disability, and Health
Rehab: Rehabilitation
