# Supplementary material for: Bridging gaps in the care of people under the age of 60 with complex impairments through cross-indication home-based rehabilitation: a cross-sectional survey with experts in Germany
Source: BMC Health Serv Res. 2026 Sep 24;26:1305. doi: 10.1186/s12913-026-15682-w (PMC13602674; doi:10.1186/s12913-026-15682-w)
Supplement: Supplementary file 1 [file 12913_2026_15682_MOESM1_ESM.pdf]

Additional file 1 Group differences on the topic "rehab services and access"

A) Group differences on the scale "unmet care needs" by professional field and knowledge of HbR

		Scale: Unmet care needs		
**Professional field**	**n[a]**	**Mdn[b] (IQR)[c]**	**P[d]**	**Effect size[e]**
Physician or senior therapist in (geriatric) HbR[g]	12	4.88 (0.41)	0.011	N/S[f]
Physician or senior therapist in cross-indication early rehab setting (i.e. hospital)	10	4.31 (1.06)		
Physician in indication-specific rehab centre	30	4.81 (0.81)		
Physician in private practice	37	4.75 (0.88)		
Discharge manager in hospital	17	5.12 (0.75)		
Assessor from the Medical Service or Socio-Medical Service	5	5.12 (0.25)		
**Knowledge of HbR[g, h]**	**n[a]**	**M[i] (SD)[j]**	**P[k]**	**d[l] [95% CI][m]**
no	51	4.61 (0.79)	0.634	-0.088 [-0.490, 0.270]
yes	67	4.68 (0.95)		

[a] Valid cases
[b] Median
[c] Interquartile range
[d] Kruskal-Wallis test (Post-hoc tests via Wilcoxon rank-sum test see below)
[e] Effect size r of the pairwise comparisons see below
[f] Not specified
[g] Home-based rehabilitation
[h] Participants stated whether HbR was already known before the survey
[i] Mean
[j] Standard deviation
[k] t-test
[l] 0.2-0.5 = small effect, 0.5-0.8 = medium effect, >0.8 = large effect
[m] 95% confidence interval

Post-hoc tests on the scale "unmet care needs" by professional field

	Pairwise group comparisons				
**Group[a]**	**n[b]**	**Group**	**n**	**P**	**r[c] [95% CI][d]**
A	12	B	10	0.019	0.509 [0.200, 0.750]
A	12	C	30	0.675	0.068 [0.002, 0.330]
A	12	D	37	0.576	0.082 [0.004, 0.330]
A	12	E	17	0.190	0.248 [0.006, 0.620]
A	12	F	5	0.100	0.412 [0.050, 0.670]
B	10	C	30	<0.001	0.423 [0.200, 0.670]
B	10	D	37	0.049	0.289 [0.080, 0.510]
B	10	E	17	0.004	0.556 [0.270, 0.740]
B	10	F	5	<0.001	0.713 [0.420, 0.840]
C	30	D	37	0.174	0.167 [0.020, 0.410]
C	30	E	17	0.375	0.131 [0.010, 0.430]
C	30	F	5	0.332	0.168 [0.004, 0.380]
D	37	E	17	0.040	0.281 [0.040, 0.470]
D	37	F	5	0.073	0.280 [0.006, 0.490]
E	17	F	5	0.875	0.042 [0.020, 0.400]

[a] A: Physician or senior therapist in (geriatric) home-based rehab, B: Physician or senior therapist in cross-indication early rehab setting (i.e. hospital), C: Physician in indication-specific rehab centre, D: Physician in private practice, E: Discharge manager in hospitals, F: Assessor from the Medical Service or Socio-Medical Service
[b] Valid cases
[c] 0.1-0.3 = small effect, 0.3-0.5 = medium effect, >0.5 = large effect
[d] 95% confidence interval

B) Group differences in further aspects of care (item level) by professional field and knowledge of HbR

**Items**	The therapeutic success achieved in outpatient rehabilitation centres can be better transferred to the familiar environment (e.g. own home) than in inpatient rehabilitation centres.				In indication-specific outpatient and inpatient rehabilitation centres, the cross-indication needs of this group of people can be clarified very well consultatively.				In outpatient and inpatient indication-specific rehabilitation centres, accompanying persons (e.g. relatives) are not sufficiently involved in the treatment concept.			
**Professional field**	**n**[a]	**Mdn**[b] **(IQR)**[c]	**P**[d]	**Effect size**[e]	**n**	**Mdn (IQR)**	**p**	**Effect size**	**n**	**Mdn (IQR)**	**p**	**Effect size**
Physician or senior therapist in (geriatric) HbR[g]	15	6.00 (1.00)	<0.001	N/S[f]	13	3.00 (1.00)	0.278	N/S	14	5.00 (1.00)	0.140	N/S
Physician or senior therapist in cross-indication early rehab setting (i.e. hospital)	10	3.00 (1.75)			11	3.00 (2.00)			11	4.00 (2.00)		
Physician in indication-specific rehab centre	34	3.00 (2.00)			33	3.00 (2.00)			31	4.00 (2.00)		
Physician in private practice	38	4.00 (2.00)			34	3.00 (1.00)			33	4.00 (1.00)		
Discharge manager in hospital	15	4.00 (1.00)			15	3.00 (1.00)			14	4.50 (1.75)		
Assessor from the Medical Service or Socio-Medical Service	5	4.00 (2.00)			5	2.00 (1.00)			5	3.00 (0.00)		
**Knowledge of HbR**[g, h]	**n**[a]	**M**[i] **(SD)**[j]	**P**[k]	**d**[l] **[95% CI]**[m]	**n**	**M (SD)**	**p**	**d [95% CI]**	**n**	**M (SD)**	**p**	**d [95% CI]**
no	55	3.45 (1.20)	<0.001	-0.619 [-1.00, -0.250]	53	3.17 (1.05)	0.244	-0.214 [-0.590, 0.140]	48	4.08 (0.99)	0.734	-0.064 [-0.47, 0.320]
yes	67	4.28 (1.46)			65	3.43 (1.37)			66	4.15 (1.14)		

[a] Valid cases
[b] Median
[c] Interquartile range
[d] Kruskal-Wallis test (if p<0.05, the post-hoc tests using the Wilcoxon rank-sum test can be viewed below)
[e] If p<0.05, the effect size of the pairwise comparisons can be viewed below
[f] Not specified
[g] Home-based rehabilitation
[h] Participants stated whether HbR was already known before the survey
[i] Mean
[j] Standard deviation
[k] t-test
[l] 0.2-0.5 = small effect, 0.5-0.8 = medium effect, >0.8 = large effect
[m] 95% confidence interval

Post-hoc tests of the item "The therapeutic success achieved…" by professional field

Pairwise group comparisons					
**Group**[a]	**n**[b]	**Group**	**n**	**p**	**r[c] [95% CI][d]**
A	15	B	10	<0.001	0.773 [0.480, 0.880]
A	15	C	34	<0.001	0.602 [0.410, 0.750]
A	15	D	38	<0.001	0.502 [0.230, 0.710]
A	15	E	15	0.002	0.581 [0.300, 0.840]
A	15	F	5	0.027	0.505 [0.080, 0.840]
B	10	C	34	0.989	0.004 [0.004, 0.300]
B	10	D	38	0.056	0.278 [0.004, 0.510]
B	10	E	15	0.012	0.507 [0.260, 0.780]
B	10	F	5	0.658	0.131 [0.020, 0.690]
C	34	D	38	0.012	0.297 [0.003, 0.540]
C	34	E	15	0.005	0.403 [0.140, 0.600]
C	34	F	5	0.651	0.076 [0.002, 0.440]
D	38	E	15	0.404	0.116 [0.007, 0.340]
D	38	F	5	0.545	0.095 [0.004, 0.420]
E	15	F	5	0.307	0.239 [0.000, 0.720]

[a] A: Physician or senior therapist in (geriatric) home-based rehab, B: Physician or senior therapist in cross-indication early rehab setting (i.e. hospital), C: Physician in indication-specific rehab centre, D: Physician in private practice, E: Discharge manager in hospitals, F: Assessor from the Medical Service or Socio-Medical Service

[b] Valid cases

[c] 0.1-0.3 = small effect, 0.3-0.5 = medium effect, >0.5 = large effect

[d] 95% confidence interval
