# Supplementary material for: Bridging gaps in the care of people under the age of 60 with complex impairments through cross-indication home-based rehabilitation: a cross-sectional survey with experts in Germany
Source: BMC Health Serv Res. 2026 Sep 24;26:1305. doi: 10.1186/s12913-026-15682-w (PMC13602674; doi:10.1186/s12913-026-15682-w)
Supplement: Supplementary file 2 [file 12913_2026_15682_MOESM2_ESM.pdf]

Additional file 2 Group differences on the topic "home care and nursing homes"

Group differences (item level) by professional field and knowledge of HbR

**Items**	Despite the need for rehabilitation, this group of people is often discharged to another setting (e.g. their own home or a nursing home) after acute inpatient care.				For this group of people, the need for rehabilitation cannot (completely) covered by the established outpatient services of private practices in occupational, speech or physiotherapy or other allied health professionals.			
**Professional field**	**n[a]**	**Mdn[b] (IQR)[c]**	**P[d]**	**Effect size[e]**	**n**	**Mdn (IQR)**	**p**	**Effect size**
Physician or senior therapist in (geriatric) HbR[g]	15	5.00 (0.00)	0.085	N/S[f]	15	5.00 (1.00)	0.463	N/S
Physician or senior therapist in cross-indication early rehab setting (i.e. hospital)	11	5.00 (2.00)			11	5.00 (1.00)		
Physician in indication-specific rehab centre	30	5.00 (1.75)			32	5.00 (2.00)		
Physician in private practice	37	5.00 (1.00)			39	5.00 (1.50)		
Discharge manager in hospital	21	5.00 (2.00)			19	5.00 (1.50)		
Assessor from the Medical Service or Socio-Medical Service	5	5.00 (1.00)			5	5.00 (2.00)		
**Knowledge of HbR[, h]**	**n[a]**	**M[i] (SD)[j]**	**P[k]**	**d[l] [95% CI][m]**	**n**	**M (SD)**	**p**	**d [95% CI]**
no	58	4.52 (0.98)	0.174	-0.244 [-0.640, 0.08]	58	4.78 (0.92)	0.237	-0.208 [-0.610, 0.120]
yes	68	4.76 (1.05)			70	5.00 (1.22)		

[a] Valid cases
[b] Median
[c] Interquartile range
[d] Kruskal-Wallis test
[e] Since p>0.05, no calculation of the effect size r of the pairwise comparisons
[f] Not specified
[g] Home-based rehabilitation
[h] Participants stated whether HbR was already known before the survey
[i] Mean
[j] Standard deviation
[k] t-test
[l] 0.2-0.5 = small effect, 0.5-0.8 = medium effect, >0.8 = large effect
[m] 95% confidence interval
