# Supplementary material for: Bridging gaps in the care of people under the age of 60 with complex impairments through cross-indication home-based rehabilitation: a cross-sectional survey with experts in Germany
Source: BMC Health Serv Res. 2026 Sep 24;26:1305. doi: 10.1186/s12913-026-15682-w (PMC13602674; doi:10.1186/s12913-026-15682-w)
Supplement: Supplementary file 3 [file 12913_2026_15682_MOESM3_ESM.pdf]

Additional file 3 Group differences on the topic "HbR in general"

Group differences on the scale "general assessment of HbR" by professional field and knowledge of HbR

		Scale: General assessment of HbR		
**Professional field**	**n**[a]	**Mdn**[b] **(IQR)**[c]	**p**[d]	**Effect size**[e]
Physician or senior therapist in (geriatric) HbR[g]	15	5.45 (0.49)	<0.001	N/S[f]
Physician or senior therapist in cross-indication early rehab setting (i.e. hospital)	10	4.48 (0.60)		
Physician in indication-specific rehab centre	30	4.82 (0.98)		
Physician in private practice	34	4.91 (0.74)		
Discharge manager in hospital	22	5.25 (0.63)		
Assessor from the Medical Service or Socio-Medical Service	4	4.35 (1.06)		
**Knowledge of HbR**[g, h]	**n**[a]	**M**[i] **(SD)**[j]	**p**[k]	**d**[l] **[95% CI]**[m]
no	53	4.87 (0.58)	0.420	-0.144 [-0.520, 0.210]
yes	68	4.99 (0.99)		

[a] Valid cases
[b] Median
[c] Interquartile range
[d] Kruskal-Wallis test (Post-hoc tests via Wilcoxon rank-sum test see below)
[e] Effect size r of the pairwise comparisons see below
[f] Not specified
[g] Home-based rehabilitation
[h] Participants stated whether HbR was already known before the survey
[i] Mean
[j] Standard deviation
[k] t-test
[l] 0.2-0.5 = small effect, 0.5-0.8 = medium effect, >0.8 = large effect
[m] 95% confidence interval

Post-hoc tests on the scale "general assessment of HbR" by professional field

Pairwise group comparisons					
**Group**[a]	**n**[b]	**Group**	**n**	**p**	**r**[c] **[95% CI]**[d]
A	15	B	10	0.002	0.622 [0.250, 0.840]
A	15	C	30	0.004	0.428 [0.180, 0.680]
A	15	D	34	0.002	0.441 [0.210, 0.640]
A	15	E	22	0.113	0.263 [0.030, 0.650]
A	15	F	4	0.008	0.622 [0.290, 0.820]
B	10	C	30	0.276	0.178 [0.004, 0.510]
B	10	D	34	0.113	0.241 [0.009, 0.550]
B	10	E	22	0.009	0.468 [0.140, 0.740]
B	10	F	4	0.671	0.132 [0.000, 0.710]
C	30	D	34	0.459	0.093 [0.004, 0.310]
C	30	E	22	0.040	0.285 [0.060, 0.540]
C	30	F	4	0.199	0.225 [0.010, 0.500]
D	34	E	22	0.060	0.253 [0.020, 0.500]
D	34	F	4	0.113	0.247 [0.020, 0.530]
E	22	F	4	0.025	0.447 [0.140, 0.640]

[a] A: Physician or senior therapist in (geriatric) home-based rehab, B: Physician or senior therapist in cross-indication early rehab setting (i.e. hospital), C: Physician in indication-specific rehab centre, D: Physician in private practice, E: Discharge manager in hospitals, F: Assessor from the Medical Service or Socio-Medical Service
[b] Valid cases
[c] 0.1-0.3 = small effect, 0.3-0.5 = medium effect, >0.5 = large effect
[d] 95% confidence interval
