# Supplementary material for: Bridging gaps in the care of people under the age of 60 with complex impairments through cross-indication home-based rehabilitation: a cross-sectional survey with experts in Germany
Source: BMC Health Serv Res. 2026 Sep 24;26:1305. doi: 10.1186/s12913-026-15682-w (PMC13602674; doi:10.1186/s12913-026-15682-w)
Supplement: Supplementary file 4 [file 12913_2026_15682_MOESM4_ESM.pdf]

Additional file 4 Group differences on the topic "cross-indication approach"

Group differences on the scale "cross-indication approach" by professional field and knowledge of HbR

	Scale: Cross-indication approach			
**Professional field**	**n**[a]	**Mdn**[b] **(IQR)**[c]	**p**[d]	**Effect size**[e]
Physician or senior therapist in (geriatric) HbR[g]	15	5.25 (0.88)	0.481	N/S[f]
Physician or senior therapist in cross-indication early rehab setting (i.e. hospital)	8	4.50 (1.38)		
Physician in indication-specific rehab centre	28	5.00 (1.56)		
Physician in private practice	35	5.00 (0.88)		
Discharge manager in hospital	20	5.00 (1.00)		
Assessor from the Medical Service or Socio-Medical Service	5	5.00 (1.00)		
**Knowledge of HbR**[g, h]	**n**[a]	**M**[i] **(SD)**[j]	**p**[k]	**d**[l] **[95% CI]**[m]
no	51	4.96 (0.80)	0.541	-0.113 [-0.520, 0.220]
yes	65	5.07 (1.04)		

[a] Valid cases
[b] Median
[c] Interquartile range
[d] Kruskal-Wallis test
[e] Since p>0.05, no calculation of the effect size r of the pairwise comparisons
[f] Not specified
[g] Home-based rehabilitation
[h] Participants stated whether HbR was already known before the survey
[i] Mean
[j] Standard deviation
[k] t-test
[l] 0.2-0.5 = small effect, 0.5-0.8 = medium effect, >0.8 = large effect
[m] 95% confidence interval
